# Adenoid cystic carcinoma: emerging role of translocations and gene fusions

**DOI:** 10.18632/oncotarget.11288

**Published:** 2016-08-14

**Authors:** Piotr T. Wysocki, Evgeny Izumchenko, Juliet Meir, Patrick K. Ha, David Sidransky, Mariana Brait

**Affiliations:** ^1^ Department of Otolaryngology and Head & Neck Surgery, Johns Hopkins University, School of Medicine, Baltimore, MD, USA; ^2^ Department of Otolaryngology- Head and Neck Surgery, University of California, San Francisco, CA, USA

**Keywords:** adenoid cystic carcinoma, salivary gland tumor, translocation, MYB, MYBL1

## Abstract

Adenoid cystic carcinoma (ACC), the second most common salivary gland malignancy, is notorious for poor prognosis, which reflects the propensity of ACC to progress to clinically advanced metastatic disease. Due to high long-term mortality and lack of effective systemic treatment, the slow-growing but aggressive ACC poses a particular challenge in head and neck oncology. Despite the advancements in cancer genomics, up until recently relatively few genetic alterations critical to the ACC development have been recognized. Although the specific chromosomal translocations resulting in *MYB*-*NFIB* fusions provide insight into the ACC pathogenesis and represent attractive diagnostic and therapeutic targets, their clinical significance is unclear, and a substantial subset of ACCs do not harbor the *MYB*-*NFIB* translocation. Strategies based on detection of newly described genetic events (such as *MYB* activating super-enhancer translocations and alterations affecting another member of MYB transcription factor family-*MYBL1*) offer new hope for improved risk assessment, therapeutic intervention and tumor surveillance. However, the impact of these approaches is still limited by an incomplete understanding of the ACC biology, and the manner by which these alterations initiate and drive ACC remains to be delineated. This manuscript summarizes the current status of gene fusions and other driver genetic alterations in ACC pathogenesis and discusses new therapeutic strategies stemming from the current research.

## INTRODUCTION

Recurrent chromosomal translocations and resultant gene fusions have long been recognized as critical events in the oncogenesis of hematological malignancies and soft-tissue neoplasms. The important role of recurrent gene fusions in epithelial malignancies started to emerge recently with the advent of new genome-wide profiling technologies. It is now recognized that actionable gene fusions are prevalent among carcinomas with over 9,100 documented translocations in solid tumors and over 176 of these described as recurrent events [1]. Although *PLAG1* rearrangements characterizing pleomorphic adenoma have been known for almost two decades [2]*,* the role of recurrent chromosomal aberrations in other types of salivary gland tumors had not been understood until now.

Recent advances such as the identification of *MECT1-MAML2* fusion in mucoepidermoid carcinoma (MEC), the recognition of a new disease entity (mammary analogue secretory carcinoma [MASC]) characterized by the *ETV6-NTRK3* fusion gene and the discovery of the *MYB-NFIB* oncogene in adenoid cystic carcinoma (ACC), have begun to refine our knowledge of salivary gland carcinogenesis [3]. ACC, one of the most common salivary gland malignancies, represents a significant challenge in head and neck oncology due to its aggressive and unpredictable phenotype. Given the high rate of late local recurrence and distant metastasis, ACC patients require intensive oncological surveillance. Unfortunately, with no effective systemic therapy available, the long-term disease control is poor and overall disease-associated mortality remains high. The discovery of the translocation between chromosome 6q and 9p and the identification of the resultant *MYB-NFIB* fusion in 2009, led to an important insight into the molecular pathogenesis of this malignancy and highlighted the tumor driving role of the *MYB* (myeloblastosis) proto-oncogene [4]. Recently, the identification of chromosomal rearrangements that juxtapose super-enhancers to the *MYB locus* and create a positive feedback elicited by activation of these enhancers by MYB protein, has further enhanced our understanding of the biology of tumors that do not harbor chimeric *MYB* transcripts [5]. Finally, the identification of a fusion between *MYBL1* and *NFIB* genes in tumors without *MYB* aberration [6, 7], demonstrates that the pathogenesis of ACC may be driven by genetic alterations in another member of the same transcription factor (TF) gene family. Although the MYB and MYBL1 fusion oncoproteins emerge as attractive diagnostic markers and therapeutic targets to improve clinical management of this lethal disease, the manner by which these and other genetic alterations initiate and drive ACC progression is not yet fully understood, and their impact on clinical outcomes remains to be delineated.

In this review, we summarize the current status of the genomic translocations in ACC, discuss challenges associated with underpinning their role in ACC pathogenesis and focus on possible clinical implications stemming from the current research.

## ADENOID CYSTIC CARCINOMA - ENIGMATIC AND CHALLENGING MALIGNANCY

ACC was first recognized as a distinct head and neck neoplasm over 150 years ago by Robin, Lorain and Laboulbene, who provided its microscopic description [8]. With an incidence of 4.5 cases per million individuals, it is the most common malignant tumor of minor salivary glands and the second most prevalent cancer of parotid and sublingual salivary glands [9, 10]. ACC arises sporadically in other exocrine glands located in breast, lacrimal glands, nasal passages, tracheobronchial tree, prostate, cervix and vulva [9, 11-16]. Interestingly, irrespective of the site of origin, these tumors display similar histological characteristics and share nonrandom cytogenetic anomalies such as copy number alterations involving chromosomes 12q, 6q, 8q, 9p, 1p and 22q [17-19]. Histologically, these neoplasms are composed of two types of cells, inner epithelial/luminal and outer myoepithelial cells, recapitulating the structure of intercalated ducts of secretory glands from which ACC are thought to originate. ACC can be classified into three subtypes: tubular and cribiform variants, which are characterized by the presence of both epithelial and myoepithelial components and display an indolent growth pattern, and the solid phenotype, associated with the loss of myoepithelial cells and more aggressive biology[20].

The clinical behavior of head and neck ACC has been described as a “paradox” [8]. While the primary tumor often manifests itself as a small and inconspicuous nodule with low growth kinetics, the disease displays a relentlessly progressive course. Consequently, although a patient's short term prognosis is favorable with an expected 5-year survival rate of 77%, rates drop significantly after 10 and 15 years with survival estimated at 60% and 45% respectively, and most patients dying as a result of the disease progression in later decades [21]. Additionally, although total resection with a clean surgical margin is usually possible, late local relapses are likely to occur even after a radical resection and adjuvant radiation therapy [11]. The ACC tumors exhibits a high tendency for neurotropic invasion leading to deep and destructive infiltration of craniofacial region, skull base and intracranial cavity [11]. Distant metastases are frequently observed in lung, liver and bones as a result of hematologic spread, while metastasis to lymph nodes are rare. Owing to these highly aggressive characteristics, it is not surprising that salivary gland ACC has long been recognized as “one of the most biologically destructive and unpredictable tumors of the head and neck” [22]. Interestingly, ACC localized in the breast exhibits favorable clinical characteristics with an excellent prognosis, despite a common growth pattern, histology and overlapping chromosomal alterations [23].

Until recently, little was known about the molecular background of the ACC's pathogenesis. Non-random chromosomal aberrations were observed and reported in clinical material since the 1980s, with special attention given to the most recurrent t(6;9) rearrangements [24-26]. Unfortunately, past efforts to identify the significance of this anomaly have been largely hampered by the lack of validated ACC cell lines [27]. In 2009, Persson et al. used short-lived primary cultures obtained from fresh tumor specimens to demonstrate that the t(6;9)(q22-23;p23-24) translocation results in a fusion between two TF genes, *MYB* and *NFIB* [4]. Although this initial study suggested that *MYB*-*NFIB* fusion might constitute a hallmark of all ACC tumors, studies that followed have detected that approximately 50% of the ACC patients do not harbor the *MYB-NFIB* translocation [15, 16, 28-31]. These studies, however, helped to refine the understanding of *MYB-NFIB* fusion oncogene as a specific and common driver of ACC pathogenesis in multiple anatomical locations, including breast [15, 30], lacrimal glands [16] and skin [32, 33]. Furthermore, overexpression of the 5′ fragment of *MYB* was observed in 89-97% [17, 34] of all ACC cases, indicating that *MYB-NFIB* fusion is not the only mechanism of MYB overexpression and suggests that ACC may also arise from other molecular aberrations involving the MYB transcription factor. Indeed, the discovery of recurrent alternate rearrangements that repose super-enhancers in the *NFIB* and *TGFBR3 loci* into proximity of *MYB* gene, uncovers an additional mechanism that may drive MYB overexpression in ACC tumors that do not express the fusion transcript [5]. Another subset of ACC tumors was found to harbor a novel *MYBL1-NFIB* fusion, an alteration found to be mutually exclusive of the “classical” *MYB-NFIB* rearrangement [6, 7]. The extensive homology in the DNA binding domain between *MYB* and *MYBL1* and a common change in the gene expression signature induced by these fusions strongly suggest that pathogenesis of virtually all of ACC tumors is uniquely driven by overexpression of members of the *MYB* TF gene family [6].

## THE ROLE OF MYB TRANSCRIPTION FACTOR FAMILY IN TUMORIGENESIS

*MYB*, one of the earliest identified proto-oncogenes, was discovered almost 30 years ago as a cellular homologue of the viral oncogene (v-MYB) carried by two different avian leukemia retroviruses, the avian acute leukemia virus (AMV) and the E26 virus [35]. MYB is a founding member of c-MYB TF family, encompassing structurally related MYBL1 (AMYB) and MYBL2 (BMYB) proteins. It plays a key role in the control of cell proliferation, survival, differentiation and angiogenesis [35, 36]. Over 80 genes are known as MYB cellular targets, including pro-proliferative genes *MYC, CCNA1, CCNB1, CCNE1, c-KIT*, anti-apoptotic *BCL-2, HSPA5, HSP70,* pro-inflammatory *COX-2* and differentiation regulator genes such as *GATA3* [35]. *MYB* has a vital functional role in the establishment of definitive hematopoiesis, inducing both expansion and differentiation of progenitor cells of erythroid and lymphoid lineages. Additionally, *MYB* drives renewal of colonic epithelium, regulates airway epithelial cells differentiation and is a critical player of adult brain neurogenesis [35, 37].

Ample evidence demonstrates that aberrant MYB expression is a potent driver of neoplasia in animal and human malignancies. The first evidence of its oncogenic potential came from the discovery that viral oncogene v-MYB is capable of inducing myeloblastic transformation in chickens and quails [35]. v-MYB represents a truncated version of the *MYB* genes and its leukemogenic potential has been linked to deletions and mutations in its C-terminal regulatory domain [38]. In humans, *MYB* overexpression is detected in most myeloid and acute lymphoid leukemia [35]. Its altered activity in hematological malignancies is often linked to the presence of recurrent chromosomal aberrations, such as amplifications, promoter rearrangements or translocations. For example, a transforming *MYB-GATA1* fusion gene has been reported in acute basophilic leukemia [39]. Furthermore, high levels of *MYB* mRNA and protein expression were detected in various solid tumors, such as colorectal and breast cancers [35]. It has been proposed that *MYB* overexpression commonly seen in these malignancies, may partially result from the disruption of the transcriptional elongation blockade imposed by the stem-loop poly-T structure formed by genomic motifs located in the first intron of *MYB* transcript. It was suggested that this stem-loop structure may lead to RNA polymerase II stalling, and subsequently result in transcription attenuation [40]. Consistent with this concept, it was reported that intronic mutations in the poly-T motifs, may reverse the transcriptional arrest and lead to increased *MYB* transcription in colon cancers [41]. In the case of breast cancer, relief of the elongation blockade has been linked to elevated activity of the estrogen receptor α [35]. Moreover, sporadic *MYB* amplifications, which have been reported in *BRCA1* positive tumors, may further contribute to the overall high levels of MYB expression in patients with breast cancer [42].

**Figure 1:**
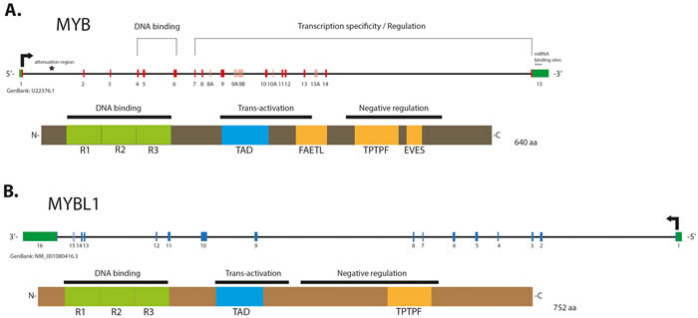
Schematic structure of gene and protein of MYB (A.) and MYBL1 (B.) Alternative exons in the genes are shown in a lighter color. The 1st intron of *MYB* contains the attenuation region, whose polyT motifs may induce formation of energetically stable stem loop which is predicted to block RNA elongation by RNA Polymerase II stalling. *MYB* contains miRNA binding sites located in its 3′-UTR and involved in repression of its transcriptional activity. MYB and MYBL1 proteins contain evolutionary conserved N-terminal R1, −2, −3 repeats forming the DNA binding domain (DBD), and a centrally located transactivation domain (TAD), both essential for the protein activity. The negative regulatory domains (NRD) are located in the C-terminal elements of the proteins. Labels indicate conserved domains: “FAETL” (which is required for oncogenic activity), “TPTF” motif (conserved in all MYB proteins) and “EVES” domain (involved in the negative regulation).

*MYB* TF consists of three functional domains: N-terminal DNA-binding domain (DBD), which recognizes a PyAACG/TG consensus sequence, a centrally located transcription activation domain (TAD); and a negative regulatory domain (NRD), located at protein's C-terminus [40] (Figure 1). Interaction of the TAD with several co-repressor and co-activator proteins, such as CBP/p300, is essential for induction and regulation of MYB transcriptional activity [40, 43]. The post-translational modifications in NRD, such as phosphorylation, acetylation, ubiquitylation and sumoylation were shown to affect MYB activity [44-47]. Studies demonstrate that NRD loss or disruption of its leucine zipper-like and EVES-motifs enhances *MYB* activity and subsequently drives neoplastic progression in several solid and hematopoietic malignancies [35, 48-50].

**Figure 2:**
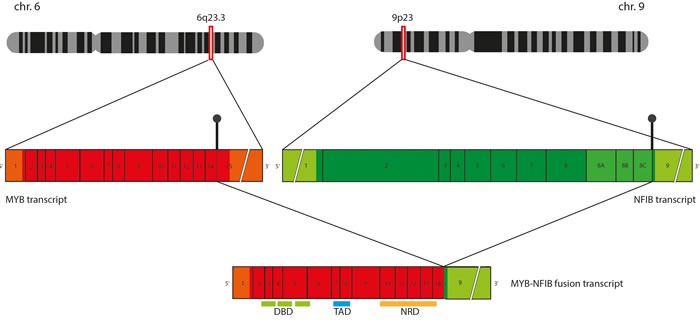
Schematic representation of *MYB-NFIB* chimeric transcript formation Translocation between chromosome 6q and 9q results in breakpoints in 3′ termini of *MYB* and *NFIB* genes, which often take place in the sequences following exon 14 of *MYB* and sequences preceding exon 9 of *NFIB*. When *MYB-NFIB* “long fusion” is formed, sequences coding the functional MYB domains (DNA binding domain [DBD], Transactivation domain [TAD] and Negative regulatory domain [NRD]) are preserved within the fusion transcript.

**Figure 3:**
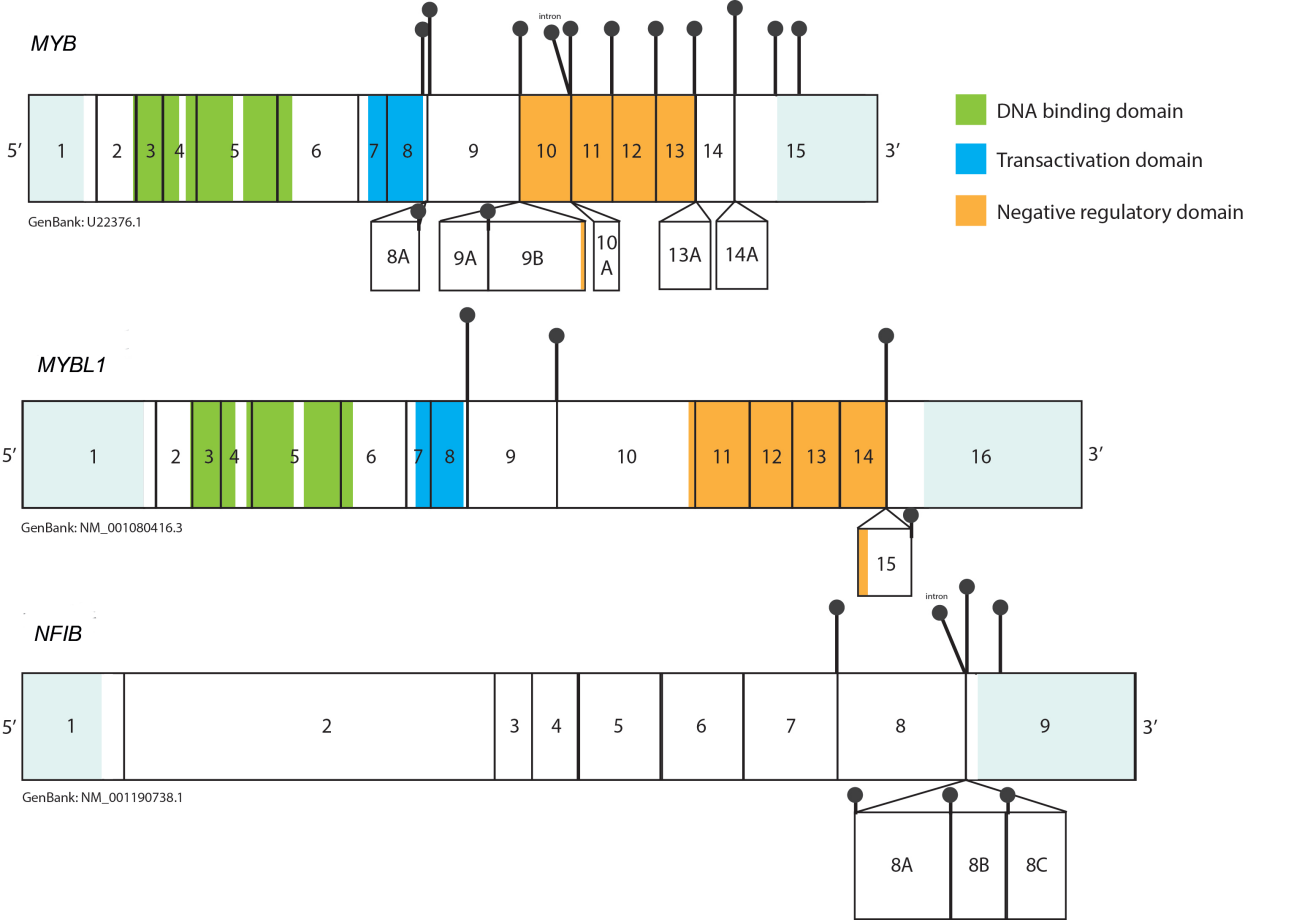
Representation of the breakpoints within the *MYB, MYBL1* and *NFIB* transcripts based on the ‘up-to-date’ literature analysis Black pins represent the breakpoints observed in the *MYB-NFIB* and *MYBL1-NFIB* transcripts. Alternative exons are shown as boxes below the main transcripts. UTRs are shown in light blue (not to scale). The breakpoint in *MYB* and *MYBL1* have been observed in sequences that follow exon 8, thus preserving DBD and TAD within all fusion oncoproteins. The proximal breakpoints (exon 8, exon 9) lead to formation of a “short fusion”, in which sequences encoding for the NRD are lost, while more distal breakpoints preserve the elements of NRD (“long fusion”). In most cases *NFIB* contributes its terminal exon 9 to the chimeric transcript. In some tumors exons 8A-8C may also be present in *MYB-NFIB* mRNA as a result of alternative splicing of *NFIB* fragment. 3′UTR breakpoints in *NFIB* have also been reported.

Involvement of *MYB* in ACC has been associated with a spectrum of complex structural rearrangements, of which, the in-frame fusion with the *NFIB* gene is the most prominent (Figure 2). Although multiple breakpoints ranging from exon 8 to 3′-UTR of the *MYB* gene have been reported in the fusion positive tumors (Figure 3), the minimal common fragment of *MYB* yet retained within the *MYB-NFIB* chimeric transcripts consists of its first 8 exons. As a result, critical functional domains of *MYB,* DBD and TAD, are always preserved within the fusion oncoprotein and contribute to its transcriptional activity [4]. In addition to the fusion of *MYB* and *NFIB*, other *MYB* translocations have been incidentally identified in ACC, including fusion of *MYB* exon 14 with intron 3 of the *PDCD1LG2* gene on chromosome 9p24 or *MYB* exon 12 with intron 22 of the *EFR3A* gene on chromosome 8q24. However, the role of these sporadic events has not yet been elucidated [29].

## MECHANISM OF *MYB* OVER EXPRESSION IN ACC

*MYB-NFIB* fusion is a dominating mechanism of 5′ *MYB* up-regulation in ACC. Persson *et al.* have postulated that *MYB* deregulation may be attributed to the miRNA target site loss [4]. The 3′ UTR of *MYB,* which is lost as a result of t(6;9)(q22-23;p23-24) translocation, contains highly conserved binding sites for certain miRNA molecules, including miR-15a, miR-16 and miR-150. It was demonstrated that transfection with these miRNAs induces a 30% down-regulation of wild-type *MYB* mRNA level in a T-cell acute lymphoblastic leukemia cell line, whereas this treatment does not decrease levels of the chimeric transcripts in ACC cells [4]. This observation is reminiscent of the mechanism observed in lipoma, where truncation of another oncogene, *HMGA2,* and its fusion with *NFIB* results in loss of miRNA target sites, which consequently leads to deregulated *HMGA2* expression [51].

Until recently, mechanisms underlying MYB overexpression in cases devoid of *MYB* structural aberrations remained largely unclear. It has been shown that epigenetic mechanisms such as promoter methylation do not play a role in regulation of *MYB* expression in ACC [52]. On the other hand, the role of *MYB*-targeting miRNAs, such as down-regulated in ACC miR-150 [53, 54], has not yet been fully elucidated. The impact of *MYB* amplification in ACC is probably limited, as copy number gains of *MYB* has been reported only incidentally [16, 55] and predominantly in cases already harboring “conventional” *MYB-NFIB* fusion [16]. The most attractive mechanism explaining MYB up-regulation in tumors without *MYB-NFIB* fusion are other critical translocations bringing regulatory elements into proximity of *MYB locus*. It has been shown that ACCs may harbor complex rearrangements, either centromeric or telomeric to *MYB locus* [17]. These structural aberrations may result in translocation of segments of chromosome 9, including sequences of the *NFIB* gene, placing them from 0.1 Mb to 10 Mb upstream of *MYB* [6, 17, 29]. This intragenic region between *MYB* and *HBS1L* contains multiple long-range enhancer elements, which bring various TFs into the proximity of *MYB* promoter and its negative regulatory elements, therefore inducing MYB expression [56, 57]. *MYB* ***locus*** is also a common site of retroviral insertion in leukemia, with multiple insertion sites localized upstream and downstream of the gene [58]. Furthermore, it has been observed that translocation of *TCRB*(T-cell receptor beta) gene to region telomeric of *MYB,* results in MYB overexpression in childhood T-cell acute lymphoblastic leukemia [58]. The hypothesis suggesting that MYB upregulation in ACC is a result of the regulatory element translocations, has been confirmed by a recent study. It has been reported that rearrangements of enhancers located within the *NFIB*, *TGFBR3* or *RAD51B loci* and their relocation upstream or downstream of the *MYB* gene, result in a significant physical interaction of these regulatory element with *MYB* promoter and subsequently high level of *MYB* mRNA expression [5]. Furthermore, binding of MYB protein to the translocated enhancers in the *NFIB* and *TGFBR3 loci*, creates a positive feedback loop that fuels further expression of *MYB*. Interestingly, the mechanisms of enhancer-driven overexpression of MYB are not limited to ACC, as an analogous event has been recently described in angiocentric gliomas, which harbor *MYB-QKI* rearrangements. It has been shown that this structural aberration drives tumorigenesis though three mechanisms: *MYB* truncation, fusion oncogene overexpression *via* translocation of enhancer elements and hemizygous loss of the *QKI* tumor suppressor [59]. Therefore, it is tempting to speculate that this “single event-multiple mechanisms” paradigm may also happen in ACC and that the relocation of *NFIB* regulatory elements contributes to high expression of the *MYB-NFIB* or *MYBL1-NFIB* chimeric transcripts in tumors that harbor the in-frame fusions between these genes.

## *MYBL1* - A PARTNER IN CRIME

*MYBL1* (*AMYB*), a gene located at chromosome 8q, is another member of the *MYB* gene family. Although MYBL1 protein and MYB share extensive structural homology in the DNA binding domain (Figure 1), and activate the same reporter genes *in vitro*, they exhibit distinct biological functions [60, 61]. In a series of deletion and domain swap experiments, Lei *et al.* have demonstrated that individual functional elements within the TAD, NRD and the C-terminus domains may play a crucial role in the target specificity of different *MYB* family members, providing a possible explanation for their diverse functionality [62]. *MYBL1* has been recently shown to be implicated in the oncogenesis of diffuse astrocytoma, which were discovered to harbor recurrent rearrangements in chromosome 8q, resulting in tandem duplication/truncation of the *MYBL1* gene. Consequently, the *MYBL1* transcript truncated at exon 9 was shown to have oncogenic properties [63, 64]. The recently discovered *MYBL1*-*NFIB* fusion gene, a result of t(8q;9p) translocation, provides another example of *MYBL1* neoplastic potential. The structure of the *MYBL1-NFIB* fusion gene exhibits a striking similarity to the *MYB-NFIB* fusion, with *MYBL1* breakpoints identified in exons 8, 9, 14 and 15, preserving the DNA binding and transactivation domains in all fusion proteins [6] (Figure 3). This fusion is mutually exclusive to the “classical” *MYB-NFIB* translocation and induces the overexpression of transcriptionally active 5′- *MYBL1* fragment [6, 7]. In a subset of ACCs, other *MYBL1* rearrangements were also observed, such as fusion with *YTHDF3* or *MYBL1* 3′- truncation, resulting in a similarly abnormal expression profile [6, 7]. Since, in the context of the fusion, both *MYB* and *MYBL1* lose elements responsible for their target specificity, the resulting oncoproteins may induce common expression signatures. Indeed, it was previously demonstrated that all ACC share similar expression profile, regardless of *MYB-NFIB* fusion status or level of *MYB* expression [53]. Common transcriptome signatures found in tumors harboring *MYB-NFIB* and *MYBL1-NFIB* fusions, further corroborate these findings [6, 7].

## IMPLICATIONS OF VARIABLE STRUCTURE OF *MYB* AND *MYBL1* IN THE FUSION TRANSCRIPTS

Past studies of the transcriptional activity of *MYB* suggest that alternately spliced RNA forms of *MYB* may produce proteins with different quantitative and qualitative activities [50]. Indeed, recent observations demonstrate that the structure of *MYB* fusion transcripts may also implicate varying level of oncogenic potential. Certain *MYB-NFIB and MYBL1-NFIB* transcripts preserve their respective NRD as a result of distal breakpoint (“long fusion”), whereas other fusion products lose it as a results of a breakpoint located in proximity of exon 8 (“short fusion”). It has been previously reported that the highest expression levels of the fusion mRNA transcripts are commonly observed in cases harboring a breakpoint in exon 8 of *MYB* [28, 29]. Recent studies suggest that “long” and “short” fusion genes may actually differ in regards to their target specificity and transcriptional activity. Mitani *et al*. [6] identified that a cohort of ACC harboring *MYB* or *MYBL1* breakpoint after exon 11 exhibits a distinct expression profile, which differs from tumors with fusions at exon 8 or 9. The first group was enriched for expression of 19 gene sets predominantly involved in RNA processing and regulation of translation, while the latter group was enriched for 5 gene sets related to tissue development [6]. Another study demonstrated that transfection with different *MYB-NFIB* and *MYBL1-NFIB* fusion genes activates the same synthetic promoters containing *MYB* binding sites, although the magnitude of activation differs significantly between fusion constructs. The transfection with the wild-type *MYB* gene or “long fusion” *MYB-NFIB* constructs containing transformation and negative regulation motifs resulted in 100-fold activation of the 5xMRE-Luc reporter, while overexpression of the “short fusion” *MYB-NFIB* or 3′-truncated *MYB* resulted in approximately 200 to 600 fold activation [7]. Although analogous findings were observed in regards to *MYBL1* and *MYBL1* fusion constructs [7], the clinical implications of these findings are unclear and warrant further investigation.

## ROLE OF *NFIB* GENE IN ACC PATHOGENESIS

Contrary to *MYB*, the role of *NFIB* (Nuclear Factor I B) in normal and cancer cell biology remains obscure. *NFIB* is a member of the Nuclear Factor I gene family, also known as ‘CAAT box TFs’ (CTF). NFIB binding sites have been identified in the promoter, enhancer and silencer elements of over 100 cellular and viral genes, although the exact function of most of them is poorly understood [65]. Studies suggest that upon dimerization and association with its target, *NFIB* may modulate transcriptional activation or repression of specific gene promoters in a tissue-specific manner [65]. The diverse cellular functions of NFIB are further corroborated by studies reporting its oncogenic or tumor suppressor roles in different tumor types. For example, *NFIB* inactivation was shown to contribute to osteosarcoma progression [66] and cutaneous carcinogenesis [67], while other studies demonstrates that *NFIB* may acts as an oncogene in small cell lung cancer [68]. A study conducted in *NFIB*-deficient mice model demonstrated that this TF plays a key role in tubule cell differentiation during embryonic development of submandibular glands [69]. Additionally, the loss of *NFIB* gene leads to fetal lung maturation defects in heterozygous *NFIB*-deficient mice, indicating possible *NFIB* haploinsufficiency [70]. Recently, it has been shown that ACC tumors, regardless of the fusion status, overexpress *NFIB* as compared to normal salivary gland tissue [71]. Although the functional significance of this upregulation is not yet clear, it may be speculated that the elevated *NFIB* expression is a result of MYB TF interaction with *NFIB* gene enhancers [5].

Although the *NFIB* fragment present within the *MYB-NFIB* fusion content may differ as a result of alternative splicing, the exon 9 (encoding the last 5 amino acids) is present in virtually all chimeric mRNA transcripts (Figure 3) [4]. It has been suggested that due to its small size, the contribution of this coding fragment to the properties of the fusion oncoprotein is likely very limited [4]. However, it is important to note that exactly the same fragment of *NFIB* is fused with *HMGA2* and *HMGIC* genes in lipoma [72] and pleomorphic adenoma [73], respectively. *HMGA2-NFIB* fusion strongly resembles the rearrangement between *MYB* and *NFIB,* as in both instances the aberration results in highly deregulated expression of DNA-binding domains of the TF linked to the small C-terminal fragment of *NFIB*. Hence, although the exact role of *NFIB* as a fusion partner remains to be discovered, it is possible that *NFIB* contributes stabilizing or regulatory elements to the fusion protein [34]. On the other hand, it has been reported that in some cases, *NFIB* fragment within the *MYB-NFIB* mRNA may be limited to its 3′ UTR only (our unpublished observations in ACC samples further support these findings) [29]. Furthermore, *NFIB* translocations have been reported in FISH t(6;9)-positive/MYB-NFIB-transcript negative ACCs, resulting in fusions between the 5′-part of *NFIB* and miscellaneous partner genes, such as *XRCC4, PTPRD, NKAIN2* or *AIG1* [6, 29]. Additionally, our lab has recently reported presence of *NFIB* fusions with the *RIMS1, MAP3K5, RPS6KA2, MYO6* genes, all of which are located on chromosome 6q [71]. It is uncertain whether these fusions produce functional proteins contributing to oncogenesis *per se*. However, it was noted that cases harboring these alternative *NFIB* gene rearrangements, have *NFIB* segments relocated into the proximity of the *MYB locus* and concurrently overexpressed the intact *MYB* transcript [6]. As discussed above, it is possible that these rearrangements relocate *NFIB*-associated super-enhancers, resulting in their physical interaction with *MYB* promoter and augmented expression of *MYB* mRNA [5].

## INCIDENCE OF THE STRUCTURAL ABERRATIONS IN ACC

The incidence of *MYB-NFIB* fusion varies across studies with reported rates ranging from 23% to 86% (Table 1). Several possible explanations for the disparities across studies may be suggested, e.g. different anatomical location of tumors tested, variable quality/origin of material studied (archival FFPE *vs*. frozen tumors), or different analytical methods used (RT-PCR, FISH, RNA-sequencing, WGS). For example, when assessed with RT-PCR, a higher incidence of *MYB-NFIB* fusions was reported in fresh-frozen material than in FFPE samples (86% *vs*. 44% respectively) [34]. Furthermore, it has been reported that positive FISH status may not always be associated with the chimeric *MYB-NFIB* transcript formation. In a subset of these ‘nontranscript forming’ tumors, breakpoints at the flanking sites of *MYB* have been identified (in many instances involving the *NFIB* segments) [5, 29]. Similarly, *NFIB* fusions with genes other than *MYB* that are located on chromosome 6q can also account for this discrepancy [71]. Consequently, our summary, which includes all up-to-date studies investigating the incidence of the *MYB-NFIB* fusion in ACC, indicates that t(6;9) rearrangement was observed in 57% (127/223) of all ACC tumors analyzed using *in situ* hybridization techniques, and the chimeric mRNA transcript was detectable in 51.1% (162/317) of the tumors (Table 1).

**Table 1:** Reported incidence of *MYB* and *MYBL1* rearrangements in ACC categorized by the detection methodology used

MYB-NFIB fusion						
Paper	ACC source	*MYB* break apart (FISH)	t(6;9) (*MYB-NFIB* FISH)	*MYB-NFIB* fusion in WGS	*MYB-NFIB* Transcript	Other structural aberrations observed in *MYB* or *NFIB*
Persson et. al 2009 [4]	Salivary, other Head and Neck, Breast		6/6		11/11	
Mitani et. al 2010, 2011, 2015 [6, 28, 29]	Salivary gland, other Head and Neck, Respiratory Tract		54/102		39/102 (RT-PCR and 3′ RACE PCR)	*MYB-PDCDILG2; MYB-EFR3A; NFIB-AIG1; NFIB-XRCC4; NFIB-NKAIN2; NFIB-PTPRD*
Brill et. al 2011 [34]	Salivary, Respiratory Tract, Breast, Vulva				39/61	
West et. al 2011 [31]	Salivary gland	24/37	18/37			*NFIB* translocation into proximity of intact *MYB; 5′ MYB* copy gain without association with *NFIB; MYB* translocation without involvement of *NFIB*
Persson et. al 2012 [17]	Salivary, Lacrimal gland, other Head and Neck, Respiratory Tract, Breast, Distant mets				30/35	*NFIB* translocation upstream of *MYB*; breakpoints distal to *MYB*
Ho et. al 2013 [74]	Salivary, Lacrimal gland, other Head and Neck, Respiratory Tract		34/60			
Costa et. al 2014 [104]	Salivary gland		3/5			
Hudson et. al 2014 [55]	Salivary gland, Respiratory Tract, Breast, Distant mets.	4/10				*MYB* trisomy without translocation in 1 case
Rettig et. al 2015 [82]	Salivary gland; other Head and Neck	59/91				
Brayer et. al 2015 [7]	Salivary gland				8/20 (RNA-seq, RT-PCR)	
Tian et. al. 2015 [105]	Cribiform salivary ACC	9/20				
Argyris et. al. 2016 [106]	Salivary gland	5/5				
Rettig et. al 2016 [71]	Salivary gland; other Head and Neck			11/25		*RIMS1-NFIB; RPS6A2-NFIB, MAP3K5-NFIB, MYO6-NFIB*
Wetterskog et. al 2012 [30]	Breast	12/13	12/13		4/13 (all negative sampes low RNA quality)	
D'Alfonso et. al 2014 [107]	Breast	7/31			6/29	
Martoletto et. al 2015 [15]	Breast	10/12			10/12	
Von Holstein et. al 2013 [16]	Lacrimal gland	8/13			7/14	*MYB* copy number gain in 1 case
Bishop et. al 2015 [108]	Prostate basal cell carcinoma	2/12 (2/7 ACC-like hist.)				
Fehr et. al 2011 [32]	Dermal cylindroma				6/11	
North et. al 2015 [33]	Primary cutaneous ACC	6/11			2/9	
Drier et. al. 2016 [5]	Primary tumors (as in Ho 2013 and Stephens 2013) and Primagrafts: Salivary gland ACC, Respiratory Tract, other Head and Neck, Distant Mets			12/20 (6 cases with loss of MYB 3′-UTR, 6 cases retaining MYB 3′-UTR)		*MYB-TGFBR3; MYB-RAD51B*; super-enhancer translocations in *NFIB, TGFBR3* and *RAD51B loci* act as drivers of *MYB* activation
TOTAL		146/250 (58.4%)	127/223 (57.0%)	23/45 (51.1%)	162/317 (51.1%)	

*MYBL1-NFIB fusion*					
Paper	ACC source	t(8;9) (*MYBL1-NFIB* FISH)	*MYBL1-NFIB* fusion in WGS	*MYBL1-NFIB* fusion transcript	Other structural aberrations in *MYBL1*
Brayer et. al 2015 [7]	Salivary gland			2/20 (RNA-seq, RT-PCR)	*MYBL1* truncation - result of *MYBL1-RAD51B* (1/20)
Mitani et. al 2015 [6]	Salivary gland, other Head and Neck, Respiratory Tract	14/102	4/21 (samples preselected with known MYB-NFIB status)	12/102	*MYBL1-YTHDF3* (2/102) *MYBL1* truncations (3/102; 1 truncation - result of *MYBL1-RAD51B*)
Drier et. al. 2016 [5]	Primary tumors (as in Ho 2013 and Stephens 2013) and Primagrafts: Salivary gland ACC, Respiratory Tract, other Head and Neck, Distant Mets		0/20		
Rettig et al. 2016 [71]	Salivary gland; other Head and Neck		2/25	2/25 (RNA-seq, RT-PCR)	
TOTAL		14/102 (13.7%)	6/66 (9.1%)	16/147 (10.9%)	6/122 (4.9%)

The second known fusion, *MYBL1*-*NFIB*, has been reported independently by three studies to be present in 11% of the ACC cases [6, 7, 71]. Our team has detected a similar incidence of this novel fusion, with 7/56 (12.5%) of ACCs identified as *MYBL1-NFIB* positive (unpublished data; manuscript in preparation). *MYBL1-NFIB* fusion was found to characterize 19% of all cases that do not harbor any *MYB* fusions*.* Additional structural aberrations involving *MYBL1*, such as fusion with *YTHDF3*, or *MYBL1* transcript truncations, have been identified in 6% of ACC tumors [6]. Taken together, the *MYB* and *MYBL1* gene rearrangements are observed in approximately two-thirds of all ACC cases.

Another subset of ACCs with no structural aberrations involving *MYB* or *MYBL1* genes, commonly overexpresses the intact *MYB* mRNA transcript. Collectively, this group contains tumors which harbor t(6;9) that do not result in formation of the *MYB-NFIB* transcript, and ACCs with no known t(6;9) translocations [6]. In the light of the recent discoveries, it appears that enhancer rearrangements in the vicinity of the *MYB locus* may account for MYB upregulation in the majority of these cases, however further studies are warranted to unravel alternative events that may also lead to *MYB* overexpression.

Comprehensive genetic studies identified low rates of somatic mutations in ACC tumors (0.3 somatic mutations per 1 Mb) with wide mutational diversity scattered among genes involved in chromatin remodeling, DNA damage/checkpoint, FGF-IGF-PI3K, Rho family, axonal guidance, Notch and MYB-MYC signaling pathways [15, 71, 74, 75]. Mutations in *NOTCH1* and *SPEN*, a negative NOTCH signaling regulator, were reported to be preferentially found in tumors with no *MYB* or *MYBL1* fusions [6]. Interestingly, another study has shown that MYB signaling cooperates with distinct pathways in eliciting biphenotypical differentiation of ACC cells, with myoepithelial cells enriched for TP63 signaling, abd Notch signal orchestrating expression program in luminal cells [5]. Absence of myopithelial component in solid histology high grade tumors has been found to be associated with activation of Notch signaling by gain-of-function mutation in *NOTCH1* or loss-of-function aberrations in *SPEN* [5]. Therefore, while dependency on Notch signaling has been associated with more aggressive phenotype, it implies that this group of ACC patients may respond to the targeted therapy with NOTCH1 inhibitors, as confirmed by a recent tumor xenograft study [76].

It is also possible that a number of cases which do not demonstrate aberrant expression of *MYB* or *MYBL1* may represent other types of salivary gland tumors with an ACC-like morphology, such as polymorphous low-grade adenocarcinoma (PLGA) or basal cell adenocarcinoma [77]. The problem of histological overlap between different salivary gland tumors may be exemplified by a report which identified an instance of *MYB-NFIB* fusion in a tumor diagnosed as PLGA [78]. However, it was recently discovered that PLGAs are characterized by a highly recurrent and pathognomonic hotspot mutation in the *PRKD1* gene [79], demonstrating that PGLA and ACC are genetically distinct entities. Future comprehensive studies may shed light on whether precise diagnosis and classification of salivary gland tumors based solely on molecular characteristics will be possible.

## IMPACT OF *MYB* AND *MYBL1* ABERRATIONS ON CLINICAL OUTCOMES

High recurrence of *MYB-NFIB* fusions and their ACC-specificity in the context of other types of head and neck neoplasms [4, 28] have encouraged various attempts to assess the diagnostic relevance of these genetic aberrations. Hudson *et al.* utilized FISH to detect *MYB* structural aberrations in cytological material obtained via fine-needle aspiration biopsy of primary and metastatic ACC tumors. Using this approach, they were able to successfully identify 5 out of 10 ACCs and distinguish them from pleomorphic adenomas (PA), which did not demonstrate *MYB* abnormalities in any of the studied cases [55]. The clinical utility for the detection of MYB protein overexpression in ACCs has also been evaluated in similar cytological material, with fine-needle aspiration biopsy specimens from ACCs found positive for MYB in most of the studied cases, successfully discriminating them from pleomorphic adenomas [80, 81].

Despite its potential diagnostic value, there is no consensus on the utility of *MYB* and *MYBL1* fusions as prognostic markers. It has been reported that *MYB-NFIB* fusion status is not significantly associated with overall survival [28, 82], although a trend toward higher likelihood of local recurrence, perineural invasion and decrease in disease-free survival (DFS) has been observed [6, 31, 82]. On the other hand, *MYB* overexpression regardless of the fusion status has been significantly associated with a poor patient survival [29]. Furthermore, combined *MYB* and *MYBL1* expression was shown to be associated with a higher disease stage and poor clinical outcome [7]. Intriguingly, a difference in the outcomes of patients with *MYB* and *MYBL1* alterations has been recently revealed, with the former group showing a significantly shorter survival [6], although studies conducted in larger cohorts of ACC patients are essential to delineate clinical impact of this finding.

## MYB TRANSCRIPTION FACTOR AS A THERAPEUTIC TARGET

Treatment options available to ACC patients remain limited to surgery and/or radiation and efficiency of currently available chemotherapeutic agents is extremely low [83]. Recurrent *MYB* alterations observed in ACC may therefore present an attractive target for potential therapeutic interventions. Unfortunately, inhibition of TF activity has been historically proven to be challenging. With no kinase activity, ligand binding sites or hydrophobic pockets that may be targeted by small molecule inhibitors (with exception of nuclear hormone receptors), inhibition of TF activity requires disruption of complex protein-DNA or protein-protein interactions [84]. Nonetheless, success in blocking protein-protein interactions, illustrated by the development of a direct NOTCH1 inhibitor [85], are encouraging and suggest that this strategy might be viable. Furthermore, it was shown that direct inhibition of other aberrant transcription factors, such as *CBFβ-SMMHC* fusion protein in AML, appears to be feasible [86].

The possibility of targeting MYB protein has predominantly been explored in leukemia studies, and several molecular approaches have been proposed (reviewed by Pattabiraman *et al.* [40]). One of these approaches suggests the use of antisense nucleotides or RNA interference to suppress *MYB* expression. Although this method proved to be successful in a mouse model of AML [87], use of RNA inference in clinical trials has been associated with substantial toxicity and problems with delivery [40]. Another approach aiming at suppression of the MYB protein expression proposes to target protein complexes that relieve the elongation blockade imposed by the poly-T motifs located in the first intron of *MYB* (see above). Deregulation of the interaction between these complexes (involving NF-kB and c-Jun) [88, 89] and the intronic region of *MYB* might constitute an attractive therapeutic strategy [40]. As MYB activity is regulated by an interplay with various partner proteins such as CBP/p300 co-activator, disruption of their physical associations might pose as another promising approach to reduce MYB activity in cancer. Interaction between the MYB transactivating domain and the KIX domain of CPB/p300 has been evaluated in depth by nuclear magnetic resonance studies [90], providing important information which may guide the development of inhibitors that could specifically bind to the protein surfaces. This strategy has been recently successfully utilized for establishment of two small molecule inhibitors of the MYB/p300 interaction, Naphthol AS-E phosphate and triterpenoid Celastrol, which were shown to impose the inhibitory effect on MYB [91, 92]. Another study was utilizing screens of small-molecule libraries and identified a promising specific inhibitor of MYB activity, natural sesquiterpene lactone mexicanin-I [93]. Although subsequent studies revealed even more potent inhibitors of *MYB* in the sesquiterpene lactone group, the exact mechanism of their action and their clinical utility remain to be delineated [94]. Finally, dependence of MYB expression on the activity of super-enhancers with strong bromodomain protein occupancy, suggests that use of BET bromodomain inhibitors may render potential anti-tumorigenic response in ACC [5]. BET inhibitors were shown to have oncostatic effect on ACC xenografts by disrupting MYB circuitry, as suggested by a modest decrease in MYB level and MYB target gene expression. This effect was however restricted to grade 2 tumors, as solid phenotype tumors (grade 3) exhibited resistance to BET inhibition, possibly reflecting their stronger dependency on NOTCH signaling, which in turn may be potentially circumvented by Notch inhibitors [5].

Targeting the downstream effectors of MYB, such as c-KIT, could pose an alternative approach to reduce MYB activity. *c-KIT* is a strong oncogene involved in leukemia, gastrointestinal stromal tumors (GIST) and melanoma [95-97], and its inhibition with imatinib has proven to be a highly successful strategy for management of these diseases. Unfortunately, although c-KIT overexpression is observed in 95% of ACCs [98], clinical trials with imatinib or second generation c-KIT inhibitors, such as dasatinib, produced no objective responses in ACC patients [83]. This has been attributed to the lack of *c-KIT* gain-of-functions mutations (which are vital for c-KIT overexpression in GIST) among the ACC tumors [20, 99]. BCL-2, a key pro-survival molecule and a MYB target, is also overexpressed in a vast majority of ACCs [98]. The development of selective BCL-2 inhibitors [100, 101] may open up a fertile avenue for novel therapeutic opportunities, but their utility in ACC has not yet been evaluated. Although inhibitors of other MYB downstream targets, such as COX-2, are currently available [98], given the broad and complex transcriptional activity of MYB, it is unlikely that targeting a single effector molecule will emerge as a successful strategy.

## CONCLUSIONS

The identification of recurring t(6;9) and t(8;9) chromosomal translocations resulting in *MYB-NFIB* and *MYBL1-NFIB* fusion oncogenes and the unravel of the role of super-enhancer translocations in the oncogene activation, dramatically extend our understanding of the role of the MYB transcription factors in the pathogenesis of ACC. New findings solidify our knowledge on the involvement of the complex structural alterations in head and neck neoplasias and indicate that disruption of regulatory mechanisms may play vital roles in overexpression of these potent oncogenes. Although the exact biological consequences of these events are not yet entirely clear, the technical progress in genomic profiling and new experiment models of ACC, such as a recently developed cell line [102] and patient-derived xenografts [103], may significantly aid in uncovering the impact of these genetic events on ACC pathogenesis. Furthermore, advancements in pharmacogenetics indicate that the MYB protein, previously regarded as an “undruggable” target, may be potently inhibited with novel promising agents. In this context, there is growing hope that intensified efforts will become fruitful with the emergence of new diagnostic and therapeutic avenues that will improve clinical outcomes for ACC patients.
